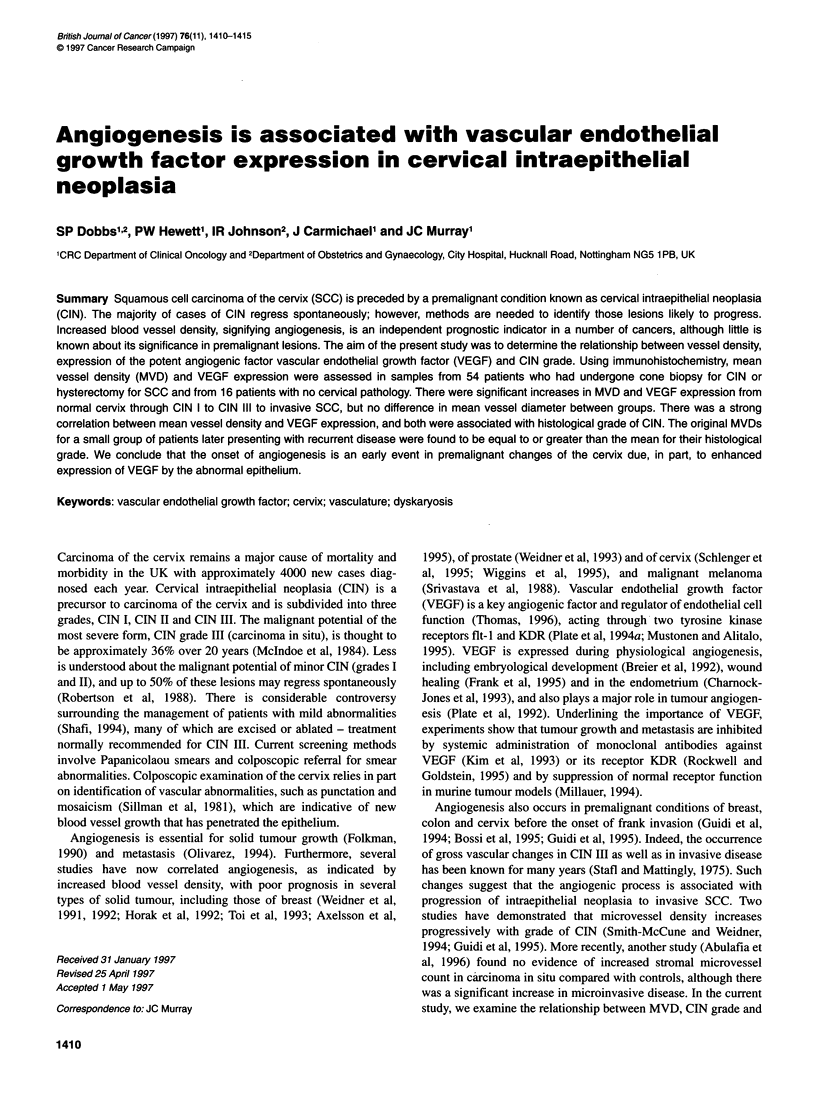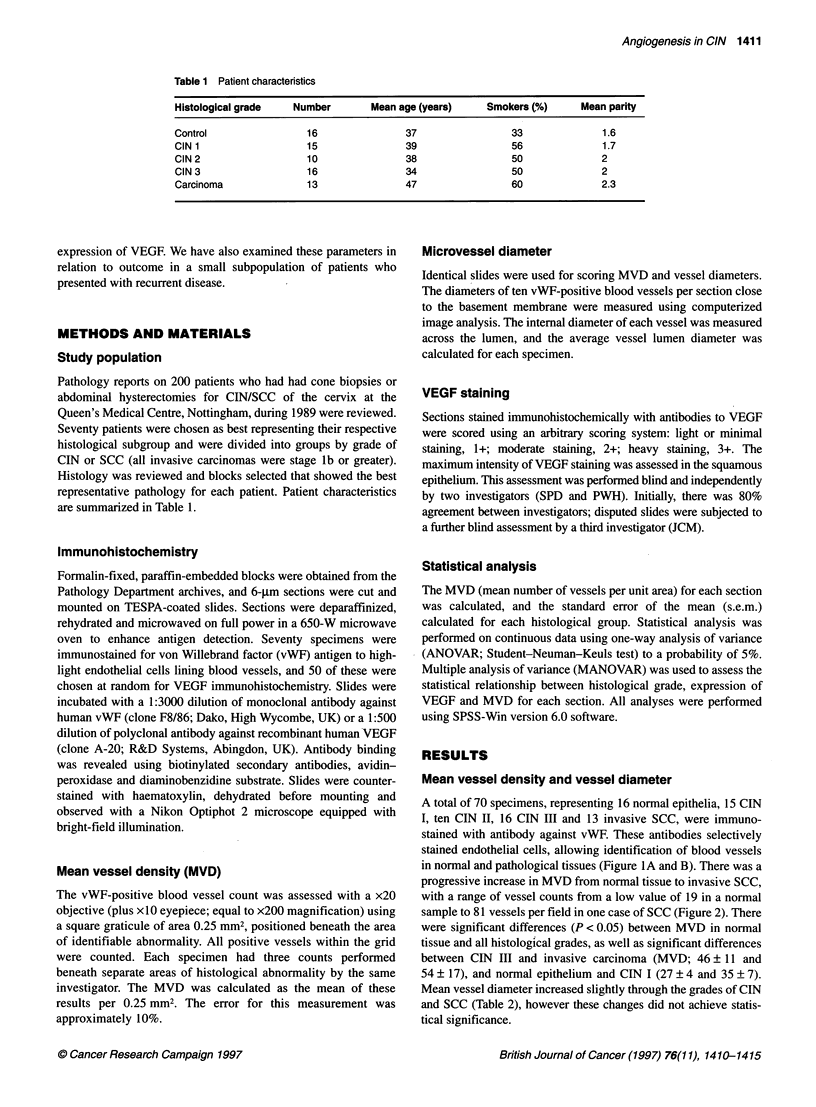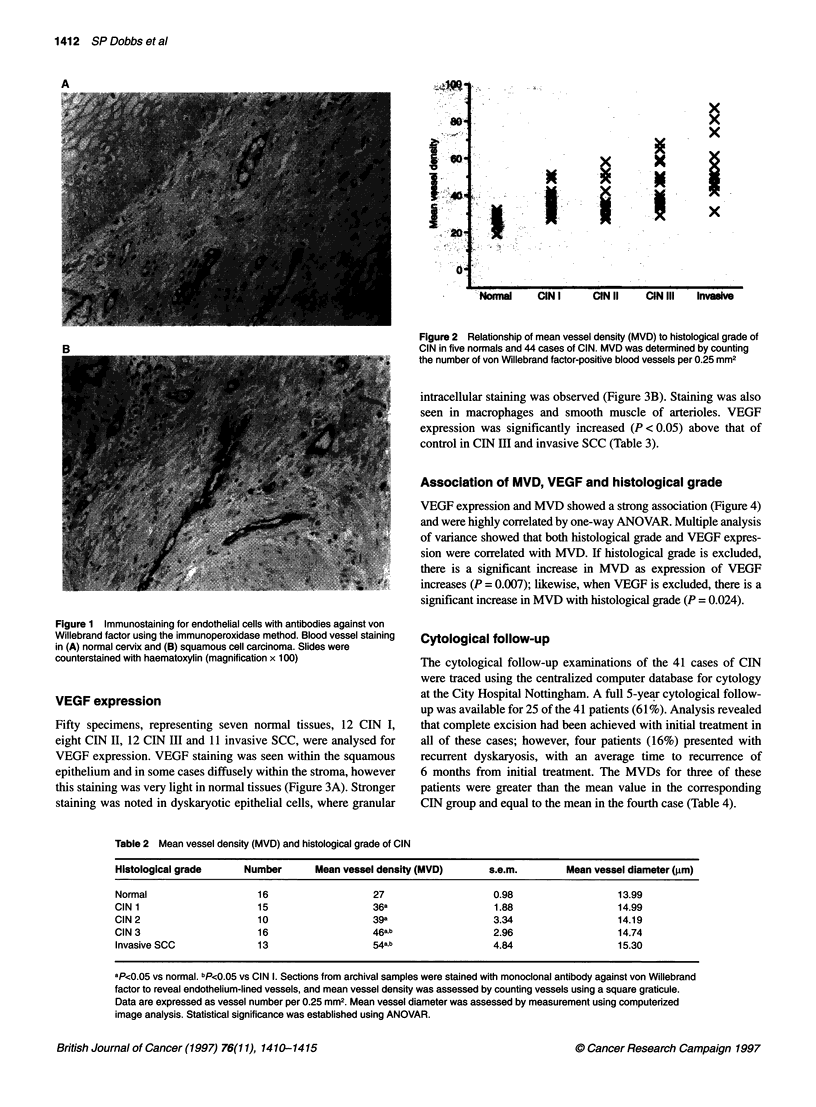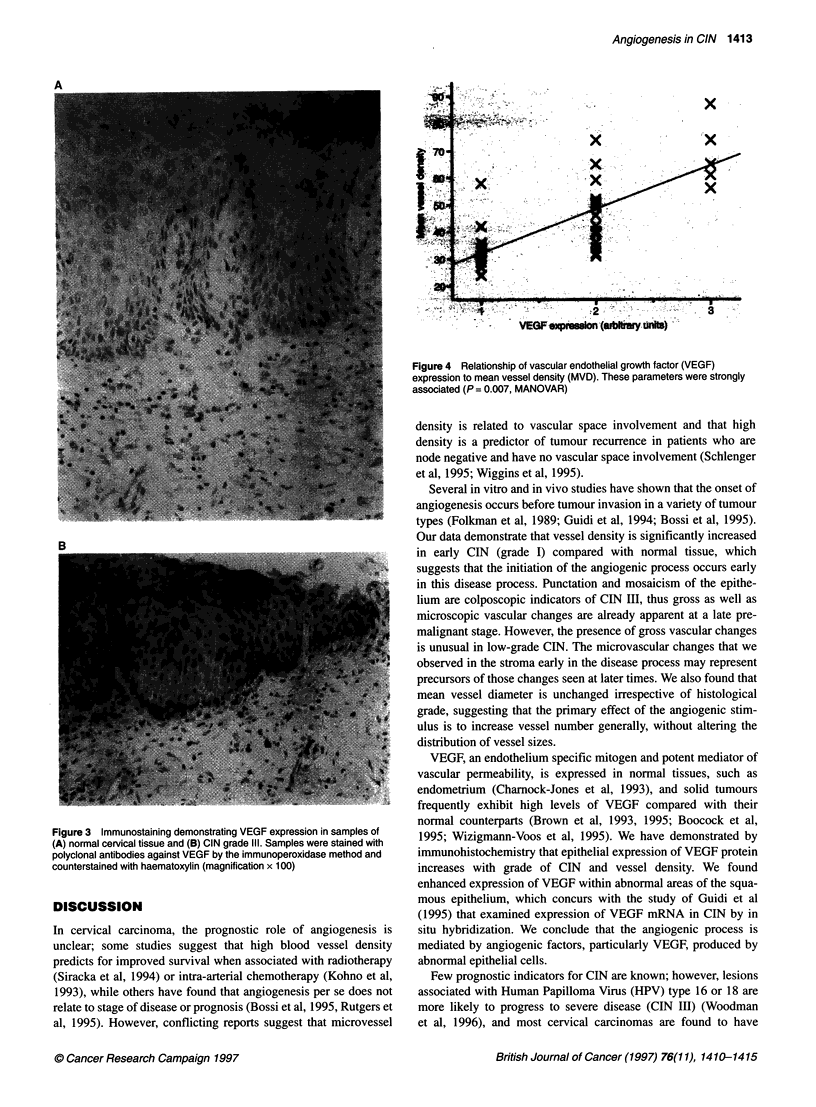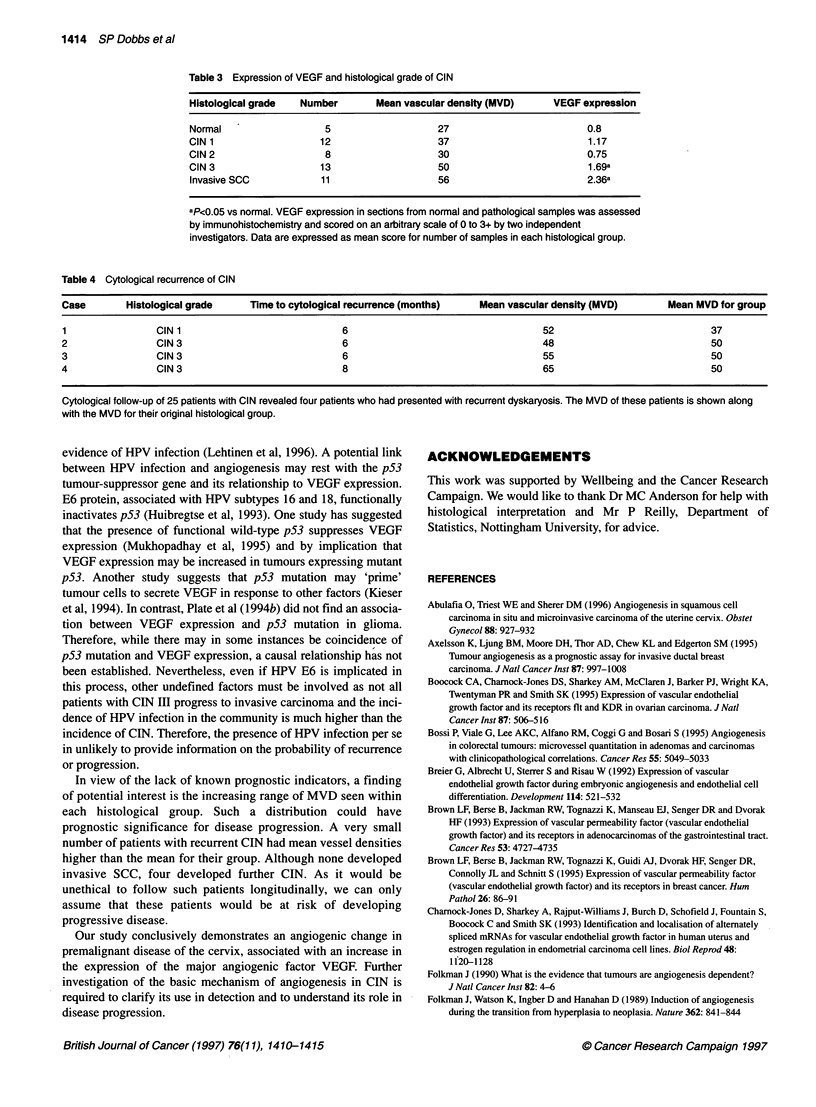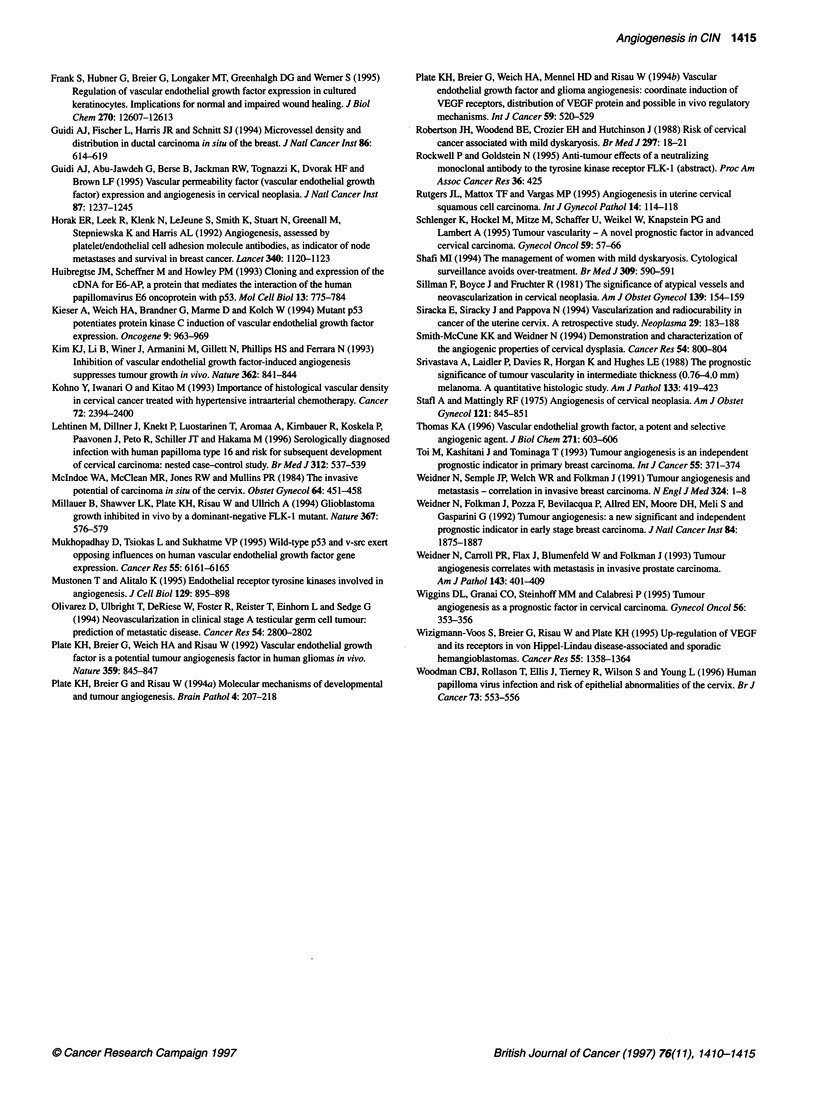# Angiogenesis is associated with vascular endothelial growth factor expression in cervical intraepithelial neoplasia.

**DOI:** 10.1038/bjc.1997.571

**Published:** 1997

**Authors:** S. P. Dobbs, P. W. Hewett, I. R. Johnson, J. Carmichael, J. C. Murray

**Affiliations:** CRC Department of Clinical Oncology, City Hospital, Nottingham, UK.

## Abstract

**Images:**


British Journal of Cancer (1997) 76(11), 1410–1415
© 1997 Cancer Research Campaign

### Angiogenesis is associated with vascular endothelial growth factor expression in cervical intraepithelial neoplasia

SP Dobbs[1,2], PW Hewett[1], IR Johnson[2], J Carmichael[1] and JC Murray[1]

[1]CRC Department of Clinical Oncology and [2]Department of Obstetrics and Gynaecology, City Hospital, Hucknall Road, Nottingham NG5 1PB, UK

**Summary** Squamous cell carcinoma of the cervix (SCC) is preceded by a premalignant condition known as cervical intraepithelial neoplasia (CIN). The majority of cases of CIN regress spontaneously; however, methods are needed to identify those lesions likely to progress. Increased blood vessel density, signifying angiogenesis, is an independent prognostic indicator in a number of cancers, although little is known about its significance in premalignant lesions. The aim of the present study was to determine the relationship between vessel density, expression of the potent angiogenic factor vascular endothelial growth factor (VEGF) and CIN grade. Using immunohistochemistry, mean vessel density (MVD) and VEGF expression were assessed in samples from 54 patients who had undergone cone biopsy for CIN or hysterectomy for SCC and from 16 patients with no cervical pathology. There were significant increases in MVD and VEGF expression from normal cervix through CIN I to CIN III to invasive SCC, but no difference in mean vessel diameter between groups. There was a strong correlation between mean vessel density and VEGF expression, and both were associated with histological grade of CIN. The original MVDs for a small group of patients later presenting with recurrent disease were found to be equal to or greater than the mean for their histological grade. We conclude that the onset of angiogenesis is an early event in premalignant changes of the cervix due, in part, to enhanced expression of VEGF by the abnormal epithelium.

**Keywords**: vascular endothelial growth factor; cervix; vasculature; dyskaryosis

Carcinoma of the cervix remains a major cause of mortality and morbidity in the UK with approximately 4000 new cases diagnosed each year. Cervical intraepithelial neoplasia (CIN) is a precursor to carcinoma of the cervix and is subdivided into three grades, CIN I, CIN II and CIN III. The malignant potential of the most severe form, CIN grade III (carcinoma in situ), is thought to be approximately 36% over 20 years (McIndoe et al, 1984). Less is understood about the malignant potential of minor CIN (grades I and II), and up to 50% of these lesions may regress spontaneously (Robertson et al, 1988). There is considerable controversy surrounding the management of patients with mild abnormalities (Shafi, 1994), many of which are excised or ablated – treatment normally recommended for CIN III. Current screening methods involve Papanicolaou smears and colposcopic referral for smear abnormalities. Colposcopic examination of the cervix relies in part on identification of vascular abnormalities, such as punctation and mosaicism (Sillman et al, 1981), which are indicative of new blood vessel growth that has penetrated the epithelium.

Angiogenesis is essential for solid tumour growth (Folkman, 1990) and metastasis (Olivarez, 1994). Furthermore, several studies have now correlated angiogenesis, as indicated by increased blood vessel density, with poor prognosis in several types of solid tumour, including those of breast (Weidner et al, 1991, 1992; Horak et al, 1992; Toi et al, 1993; Axelsson et al, 1995), of prostate (Weidner et al, 1993) and of cervix (Schlenger et al, 1995; Wiggins et al, 1995), and malignant melanoma (Srivastava et al, 1988). Vascular endothelial growth factor (VEGF) is a key angiogenic factor and regulator of endothelial cell function (Thomas, 1996), acting through two tyrosine kinase receptors flt-1 and KDR (Plate et al, 1994*a*; Mustonen and Alitalo, 1995). VEGF is expressed during physiological angiogenesis, including embryological development (Breier et al, 1992), wound healing (Frank et al, 1995) and in the endometrium (Charnock-Jones et al, 1993), and also plays a major role in tumour angiogenesis (Plate et al, 1992). Underlining the importance of VEGF, experiments show that tumour growth and metastasis are inhibited by systemic administration of monoclonal antibodies against VEGF (Kim et al, 1993) or its receptor KDR (Rockwell and Goldstein, 1995) and by suppression of normal receptor function in murine tumour models (Millauer, 1994).

Angiogenesis also occurs in premalignant conditions of breast, colon and cervix before the onset of frank invasion (Guidi et al, 1994; Bossi et al, 1995; Guidi et al, 1995). Indeed, the occurrence of gross vascular changes in CIN III as well as in invasive disease has been known for many years (Stafl and Mattingly, 1975). Such changes suggest that the angiogenic process is associated with progression of intraepithelial neoplasia to invasive SCC. Two studies have demonstrated that microvessel density increases progressively with grade of CIN (Smith-McCune and Weidner, 1994; Guidi et al, 1995). More recently, another study (Abulafia et al, 1996) found no evidence of increased stromal microvessel count in carcinoma in situ compared with controls, although there was a significant increase in microinvasive disease. In the current study, we examine the relationship between MVD, CIN grade and

*Received 31 January 1997*
*Revised 25 April 1997*
*Accepted 1 May 1997*

*Correspondence to:* JC Murray

**Table 1** Patient characteristics

| Histological grade | Number | Mean age (years) | Smokers (%) | Mean parity |
|---|---|---|---|---|
| Control | 16 | 37 | 33 | 1.6 |
| CIN 1 | 15 | 39 | 56 | 1.7 |
| CIN 2 | 10 | 38 | 50 | 2 |
| CIN 3 | 16 | 34 | 50 | 2 |
| Carcinoma | 13 | 47 | 60 | 2.3 |

expression of VEGF. We have also examined these parameters in relation to outcome in a small subpopulation of patients who presented with recurrent disease.

### METHODS AND MATERIALS

### Study population

Pathology reports on 200 patients who had had cone biopsies or abdominal hysterectomies for CIN/SCC of the cervix at the Queen's Medical Centre, Nottingham, during 1989 were reviewed. Seventy patients were chosen as best representing their respective histological subgroup and were divided into groups by grade of CIN or SCC (all invasive carcinomas were stage 1b or greater). Histology was reviewed and blocks selected that showed the best representative pathology for each patient. Patient characteristics are summarized in Table 1.

### Immunohistochemistry

Formalin-fixed, paraffin-embedded blocks were obtained from the Pathology Department archives, and 6-μm sections were cut and mounted on TESPA-coated slides. Sections were deparaffinized, rehydrated and microwaved on full power in a 650-W microwave oven to enhance antigen detection. Seventy specimens were immunostained for von Willebrand factor (vWF) antigen to highlight endothelial cells lining blood vessels, and 50 of these were chosen at random for VEGF immunohistochemistry. Slides were incubated with a 1:3000 dilution of monoclonal antibody against human vWF (clone F8/86; Dako, High Wycombe, UK) or a 1:500 dilution of polyclonal antibody against recombinant human VEGF (clone A-20; R&D Systems, Abingdon, UK). Antibody binding was revealed using biotinylated secondary antibodies, avidin–peroxidase and diaminobenzidine substrate. Slides were counterstained with haematoxylin, dehydrated before mounting and observed with a Nikon Optiphot 2 microscope equipped with bright-field illumination.

### Mean vessel density (MVD)

The vWF-positive blood vessel count was assessed with a ×20 objective (plus ×10 eyepiece; equal to ×200 magnification) using a square graticule of area 0.25 $mm^2$, positioned beneath the area of identifiable abnormality. All positive vessels within the grid were counted. Each specimen had three counts performed beneath separate areas of histological abnormality by the same investigator. The MVD was calculated as the mean of these results per 0.25 $mm^2$. The error for this measurement was approximately 10%.

### Microvessel diameter

Identical slides were used for scoring MVD and vessel diameters. The diameters of ten vWF-positive blood vessels per section close to the basement membrane were measured using computerized image analysis. The internal diameter of each vessel was measured across the lumen, and the average vessel lumen diameter was calculated for each specimen.

### VEGF staining

Sections stained immunohistochemically with antibodies to VEGF were scored using an arbitrary scoring system: light or minimal staining, 1+; moderate staining, 2+; heavy staining, 3+. The maximum intensity of VEGF staining was assessed in the squamous epithelium. This assessment was performed blind and independently by two investigators (SPD and PWH). Initially, there was 80% agreement between investigators; disputed slides were subjected to a further blind assessment by a third investigator (JCM).

### Statistical analysis

The MVD (mean number of vessels per unit area) for each section was calculated, and the standard error of the mean (s.e.m.) calculated for each histological group. Statistical analysis was performed on continuous data using one-way analysis of variance (ANOVAR; Student–Neuman–Keuls test) to a probability of 5%. Multiple analysis of variance (MANOVAR) was used to assess the statistical relationship between histological grade, expression of VEGF and MVD for each section. All analyses were performed using SPSS-Win version 6.0 software.

### RESULTS

### Mean vessel density and vessel diameter

A total of 70 specimens, representing 16 normal epithelia, 15 CIN I, ten CIN II, 16 CIN III and 13 invasive SCC, were immunostained with antibody against vWF. These antibodies selectively stained endothelial cells, allowing identification of blood vessels in normal and pathological tissues (Figure 1A and B). There was a progressive increase in MVD from normal tissue to invasive SCC, with a range of vessel counts from a low value of 19 in a normal sample to 81 vessels per field in one case of SCC (Figure 2). There were significant differences ($P < 0.05$) between MVD in normal tissue and all histological grades, as well as significant differences between CIN III and invasive carcinoma (MVD; $46 \pm 11$ and $54 \pm 17$), and normal epithelium and CIN I ($27 \pm 4$ and $35 \pm 7$). Mean vessel diameter increased slightly through the grades of CIN and SCC (Table 2), however these changes did not achieve statistical significance.

© Cancer Research Campaign 1997

Figure 1 Immunostaining for endothelial cells with antibodies against von Willebrand factor using the immunoperoxidase method. Blood vessel staining in (A) normal cervix and (B) squamous cell carcinoma. Slides were counterstained with haematoxylin (magnification × 100)

### VEGF expression

Fifty specimens, representing seven normal tissues, 12 CIN I, eight CIN II, 12 CIN III and 11 invasive SCC, were analysed for VEGF expression. VEGF staining was seen within the squamous epithelium and in some cases diffusely within the stroma, however this staining was very light in normal tissues (Figure 3A). Stronger staining was noted in dyskaryotic epithelial cells, where granular

Figure 2 Relationship of mean vessel density (MVD) to histological grade of CIN in five normals and 44 cases of CIN. MVD was determined by counting the number of von Willebrand factor-positive blood vessels per 0.25 $mm^2$

intracellular staining was observed (Figure 3B). Staining was also seen in macrophages and smooth muscle of arterioles. VEGF expression was significantly increased ($P < 0.05$) above that of control in CIN III and invasive SCC (Table 3).

### Association of MVD, VEGF and histological grade

VEGF expression and MVD showed a strong association (Figure 4) and were highly correlated by one-way ANOVAR. Multiple analysis of variance showed that both histological grade and VEGF expression were correlated with MVD. If histological grade is excluded, there is a significant increase in MVD as expression of VEGF increases ($P = 0.007$); likewise, when VEGF is excluded, there is a significant increase in MVD with histological grade ($P = 0.024$).

### Cytological follow-up

The cytological follow-up examinations of the 41 cases of CIN were traced using the centralized computer database for cytology at the City Hospital Nottingham. A full 5-year cytological follow-up was available for 25 of the 41 patients (61%). Analysis revealed that complete excision had been achieved with initial treatment in all of these cases; however, four patients (16%) presented with recurrent dyskaryosis, with an average time to recurrence of 6 months from initial treatment. The MVDs for three of these patients were greater than the mean value in the corresponding CIN group and equal to the mean in the fourth case (Table 4).

**Table 2** Mean vessel density (MVD) and histological grade of CIN

| Histological grade | Number | Mean vessel density (MVD) | s.e.m. | Mean vessel diameter (μm) |
|---|---|---|---|---|
| Normal | 16 | 27 | 0.98 | 13.99 |
| CIN 1 | 15 | 36[a] | 1.88 | 14.99 |
| CIN 2 | 10 | 39[a] | 3.34 | 14.19 |
| CIN 3 | 16 | 46[a,b] | 2.96 | 14.74 |
| Invasive SCC | 13 | 54[a,b] | 4.84 | 15.30 |

[a]$P<0.05$ vs normal. [b]$P<0.05$ vs CIN I. Sections from archival samples were stained with monoclonal antibody against von Willebrand factor to reveal endothelium-lined vessels, and mean vessel density was assessed by counting vessels using a square graticule. Data are expressed as vessel number per 0.25 $mm^2$. Mean vessel diameter was assessed by measurement using computerized image analysis. Statistical significance was established using ANOVAR.

© Cancer Research Campaign 1997

Figure 3 Immunostaining demonstrating VEGF expression in samples of (A) normal cervical tissue and (B) CIN grade III. Samples were stained with polyclonal antibodies against VEGF by the immunoperoxidase method and counterstained with haematoxylin (magnification × 100)

### DISCUSSION

In cervical carcinoma, the prognostic role of angiogenesis is unclear; some studies suggest that high blood vessel density predicts for improved survival when associated with radiotherapy (Siracka et al, 1994) or intra-arterial chemotherapy (Kohno et al, 1993), while others have found that angiogenesis per se does not relate to stage of disease or prognosis (Bossi et al, 1995, Rutgers et al, 1995). However, conflicting reports suggest that microvessel

**Figure 4** Relationship of vascular endothelial growth factor (VEGF) expression to mean vessel density (MVD). These parameters were strongly associated ($P = 0.007$, MANOVAR)

density is related to vascular space involvement and that high density is a predictor of tumour recurrence in patients who are node negative and have no vascular space involvement (Schlenger et al, 1995; Wiggins et al, 1995).

Several in vitro and in vivo studies have shown that the onset of angiogenesis occurs before tumour invasion in a variety of tumour types (Folkman et al, 1989; Guidi et al, 1994; Bossi et al, 1995). Our data demonstrate that vessel density is significantly increased in early CIN (grade I) compared with normal tissue, which suggests that the initiation of the angiogenic process occurs early in this disease process. Punctation and mosaicism of the epithelium are colposcopic indicators of CIN III, thus gross as well as microscopic vascular changes are already apparent at a late premalignant stage. However, the presence of gross vascular changes is unusual in low-grade CIN. The microvascular changes that we observed in the stroma early in the disease process may represent precursors of those changes seen at later times. We also found that mean vessel diameter is unchanged irrespective of histological grade, suggesting that the primary effect of the angiogenic stimulus is to increase vessel number generally, without altering the distribution of vessel sizes.

VEGF, an endothelium specific mitogen and potent mediator of vascular permeability, is expressed in normal tissues, such as endometrium (Charnock-Jones et al, 1993), and solid tumours frequently exhibit high levels of VEGF compared with their normal counterparts (Brown et al, 1993, 1995; Boocock et al, 1995; Wizigmann-Voos et al, 1995). We have demonstrated by immunohistochemistry that epithelial expression of VEGF protein increases with grade of CIN and vessel density. We found enhanced expression of VEGF within abnormal areas of the squamous epithelium, which concurs with the study of Guidi et al (1995) that examined expression of VEGF mRNA in CIN by in situ hybridization. We conclude that the angiogenic process is mediated by angiogenic factors, particularly VEGF, produced by abnormal epithelial cells.

Few prognostic indicators for CIN are known; however, lesions associated with Human Papilloma Virus (HPV) type 16 or 18 are more likely to progress to severe disease (CIN III) (Woodman et al, 1996), and most cervical carcinomas are found to have

© Cancer Research Campaign 1997

**Table 3** Expression of VEGF and histological grade of CIN

| Histological grade | Number | Mean vascular density (MVD) | VEGF expression |
|---|---|---|---|
| Normal | 5 | 27 | 0.8 |
| CIN 1 | 12 | 37 | 1.17 |
| CIN 2 | 8 | 30 | 0.75 |
| CIN 3 | 13 | 50 | 1.69[a] |
| Invasive SCC | 11 | 56 | 2.36[a] |

[a]*P*<0.05 vs normal. VEGF expression in sections from normal and pathological samples was assessed by immunohistochemistry and scored on an arbitrary scale of 0 to 3+ by two independent investigators. Data are expressed as mean score for number of samples in each histological group.

**Table 4** Cytological recurrence of CIN

| Case | Histological grade | Time to cytological recurrence (months) | Mean vascular density (MVD) | Mean MVD for group |
|---|---|---|---|---|
| 1 | CIN 1 | 6 | 52 | 37 |
| 2 | CIN 3 | 6 | 48 | 50 |
| 3 | CIN 3 | 6 | 55 | 50 |
| 4 | CIN 3 | 8 | 65 | 50 |

Cytological follow-up of 25 patients with CIN revealed four patients who had presented with recurrent dyskaryosis. The MVD of these patients is shown along with the MVD for their original histological group.

evidence of HPV infection (Lehtinen et al, 1996). A potential link between HPV infection and angiogenesis may rest with the *p53* tumour-suppressor gene and its relationship to VEGF expression. E6 protein, associated with HPV subtypes 16 and 18, functionally inactivates *p53* (Huibregtse et al, 1993). One study has suggested that the presence of functional wild-type *p53* suppresses VEGF expression (Mukhopadhay et al, 1995) and by implication that VEGF expression may be increased in tumours expressing mutant *p53*. Another study suggests that *p53* mutation may 'prime' tumour cells to secrete VEGF in response to other factors (Kieser et al, 1994). In contrast, Plate et al (1994*b*) did not find an association between VEGF expression and *p53* mutation in glioma. Therefore, while there may in some instances be coincidence of *p53* mutation and VEGF expression, a causal relationship has not been established. Nevertheless, even if HPV E6 is implicated in this process, other undefined factors must be involved as not all patients with CIN III progress to invasive carcinoma and the incidence of HPV infection in the community is much higher than the incidence of CIN. Therefore, the presence of HPV infection per se in unlikely to provide information on the probability of recurrence or progression.

In view of the lack of known prognostic indicators, a finding of potential interest is the increasing range of MVD seen within each histological group. Such a distribution could have prognostic significance for disease progression. A very small number of patients with recurrent CIN had mean vessel densities higher than the mean for their group. Although none developed invasive SCC, four developed further CIN. As it would be unethical to follow such patients longitudinally, we can only assume that these patients would be at risk of developing progressive disease.

Our study conclusively demonstrates an angiogenic change in premalignant disease of the cervix, associated with an increase in the expression of the major angiogenic factor VEGF. Further investigation of the basic mechanism of angiogenesis in CIN is required to clarify its use in detection and to understand its role in disease progression.

### ACKNOWLEDGEMENTS

This work was supported by Wellbeing and the Cancer Research Campaign. We would like to thank Dr MC Anderson for help with histological interpretation and Mr P Reilly, Department of Statistics, Nottingham University, for advice.

### REFERENCES

Abulafia O, Triest WE and Sherer DM (1996) Angiogenesis in squamous cell carcinoma in situ and microinvasive carcinoma of the uterine cervix. *Obstet Gynecol* **88**: 927–932

Axelsson K, Ljung BM, Moore DH, Thor AD, Chew KL and Edgerton SM (1995) Tumour angiogenesis as a prognostic assay for invasive ductal breast carcinoma. *J Natl Cancer Inst* **87**: 997–1008

Boocock CA, Charnock-Jones DS, Sharkey AM, McClaren J, Barker PJ, Wright KA, Twentyman PR and Smith SK (1995) Expression of vascular endothelial growth factor and its receptors flt and KDR in ovarian carcinoma. *J Natl Cancer Inst* **87**: 506–516

Bossi P, Viale G, Lee AKC, Alfano RM, Coggi G and Bosari S (1995) Angiogenesis in colorectal tumours: microvessel quantitation in adenomas and carcinomas with clinicopathological correlations. *Cancer Res* **55**: 5049–5033

Breier G, Albrecht U, Sterrer S and Risau W (1992) Expression of vascular endothelial growth factor during embryonic angiogenesis and endothelial cell differentiation. *Development* **114**: 521–532

Brown LF, Berse B, Jackman RW, Tognazzi K, Manseau EJ, Senger DR and Dvorak HF (1993) Expression of vascular permeability factor (vascular endothelial growth factor) and its receptors in adenocarcinomas of the gastrointestinal tract. *Cancer Res* **53**: 4727–4735

Brown LF, Berse B, Jackman RW, Tognazzi K, Guidi AJ, Dvorak HF, Senger DR, Connolly JL and Schnitt S (1995) Expression of vascular permeability factor (vascular endothelial growth factor) and its receptors in breast cancer. *Hum Pathol* **26**: 86–91

Charnock-Jones D, Sharkey A, Rajput-Williams J, Burch D, Schofield J, Fountain S, Boocock C and Smith SK (1993) Identification and localisation of alternately spliced mRNAs for vascular endothelial growth factor in human uterus and estrogen regulation in endometrial carcinoma cell lines. *Biol Reprod* **48**: 1120–1128

Folkman J (1990) What is the evidence that tumours are angiogenesis dependent? *J Natl Cancer Inst* **82**: 4–6

Folkman J, Watson K, Ingber D and Hanahan D (1989) Induction of angiogenesis during the transition from hyperplasia to neoplasia. *Nature* **362**: 841–844

© Cancer Research Campaign 1997

Frank S, Hubner G, Breier G, Longaker MT, Greenhalgh DG and Werner S (1995) Regulation of vascular endothelial growth factor expression in cultured keratinocytes. Implications for normal and impaired wound healing. *J Biol Chem* **270**: 12607–12613

Guidi AJ, Fischer L, Harris JR and Schnitt SJ (1994) Microvessel density and distribution in ductal carcinoma *in situ* of the breast. *J Natl Cancer Inst* **86**: 614–619

Guidi AJ, Abu-Jawdeh G, Berse B, Jackman RW, Tognazzi K, Dvorak HF and Brown LF (1995) Vascular permeability factor (vascular endothelial growth factor) expression and angiogenesis in cervical neoplasia. *J Natl Cancer Inst* **87**: 1237–1245

Horak ER, Leek R, Klenk N, LeJeune S, Smith K, Stuart N, Greenall M, Stepniewska K and Harris AL (1992) Angiogenesis, assessed by platelet/endothelial cell adhesion molecule antibodies, as indicator of node metastases and survival in breast cancer. *Lancet* **340**: 1120–1123

Huibregtse JM, Scheffner M and Howley PM (1993) Cloning and expression of the cDNA for E6-AP, a protein that mediates the interaction of the human papillomavirus E6 oncoprotein with p53. *Mol Cell Biol* **13**: 775–784

Kieser A, Weich HA, Brandner G, Marme D and Kolch W (1994) Mutant p53 potentiates protein kinase C induction of vascular endothelial growth factor expression. *Oncogene* **9**: 963–969

Kim KJ, Li B, Winer J, Armanini M, Gillett N, Phillips HS and Ferrara N (1993) Inhibition of vascular endothelial growth factor-induced angiogenesis suppresses tumour growth *in vivo*. *Nature* **362**: 841–844

Kohno Y, Iwanari O and Kitao M (1993) Importance of histological vascular density in cervical cancer treated with hypertensive intraarterial chemotherapy. *Cancer* **72**: 2394–2400

Lehtinen M, Dillner J, Knekt P, Luostarinen T, Aromaa A, Kirnbauer R, Koskela P, Paavonen J, Peto R, Schiller JT and Hakama M (1996) Serologically diagnosed infection with human papilloma type 16 and risk for subsequent development of cervical carcinoma: nested case–control study. *Br Med J* **312**: 537–539

McIndoe WA, McClean MR, Jones RW and Mullins PR (1984) The invasive potential of carcinoma *in situ* of the cervix. *Obstet Gynecol* **64**: 451–458

Millauer B, Shawver LK, Plate KH, Risau W and Ullrich A (1994) Glioblastoma growth inhibited in vivo by a dominant-negative FLK-1 mutant. *Nature* **367**: 576–579

Mukhopadhay D, Tsiokas L and Sukhatme VP (1995) Wild-type p53 and v-src exert opposing influences on human vascular endothelial growth factor gene expression. *Cancer Res* **55**: 6161–6165

Mustonen T and Alitalo K (1995) Endothelial receptor tyrosine kinases involved in angiogenesis. *J Cell Biol* **129**: 895–898

Olivarez D, Ulbright T, DeRiese W, Foster R, Reister T, Einhorn L and Sedge G (1994) Neovascularization in clinical stage A testicular germ cell tumour: prediction of metastatic disease. *Cancer Res* **54**: 2800–2802

Plate KH, Breier G, Weich HA and Risau W (1992) Vascular endothelial growth factor is a potential tumour angiogenesis factor in human gliomas *in vivo*. *Nature* **359**: 845–847

Plate KH, Breier G and Risau W (1994*a*) Molecular mechanisms of developmental and tumour angiogenesis. *Brain Pathol* **4**: 207–218

Plate KH, Breier G, Weich HA, Mennel HD and Risau W (1994*b*) Vascular endothelial growth factor and glioma angiogenesis: coordinate induction of VEGF receptors, distribution of VEGF protein and possible in vivo regulatory mechanisms. *Int J Cancer* **59**: 520–529

Robertson JH, Woodend BE, Crozier EH and Hutchinson J (1988) Risk of cervical cancer associated with mild dyskaryosis. *Br Med J* **297**: 18–21

Rockwell P and Goldstein N (1995) Anti-tumour effects of a neutralizing monoclonal antibody to the tyrosine kinase receptor FLK-1 (abstract). *Proc Am Assoc Cancer Res* **36**: 425

Rutgers JL, Mattox TF and Vargas MP (1995) Angiogenesis in uterine cervical squamous cell carcinoma. *Int J Gynecol Pathol* **14**: 114–118

Schlenger K, Hockel M, Mitze M, Schaffer U, Weikel W, Knapstein PG and Lambert A (1995) Tumour vascularity – A novel prognostic factor in advanced cervical carcinoma. *Gynecol Oncol* **59**: 57–66

Shafi MI (1994) The management of women with mild dyskaryosis. Cytological surveillance avoids over-treatment. *Br Med J* **309**: 590–591

Sillman F, Boyce J and Fruchter R (1981) The significance of atypical vessels and neovascularization in cervical neoplasia. *Am J Obstet Gynecol* **139**: 154–159

Siracka E, Siracky J and Pappova N (1994) Vascularization and radiocurability in cancer of the uterine cervix. A retrospective study. *Neoplasma* **29**: 183–188

Smith-McCune KK and Weidner N (1994) Demonstration and characterization of the angiogenic properties of cervical dysplasia. *Cancer Res* **54**: 800–804

Srivastava A, Laidler P, Davies R, Horgan K and Hughes LE (1988) The prognostic significance of tumour vascularity in intermediate thickness (0.76–4.0 mm) melanoma. A quantitative histologic study. *Am J Pathol* **133**: 419–423

Stafl A and Mattingly RF (1975) Angiogenesis of cervical neoplasia. *Am J Obstet Gynecol* **121**: 845–851

Thomas KA (1996) Vascular endothelial growth factor, a potent and selective angiogenic agent. *J Biol Chem* **271**: 603–606

Toi M, Kashitani J and Tominaga T (1993) Tumour angiogenesis is an independent prognostic indicator in primary breast carcinoma. *Int J Cancer* **55**: 371–374

Weidner N, Semple JP, Welch WR and Folkman J (1991) Tumour angiogenesis and metastasis – correlation in invasive breast carcinoma. *N Engl J Med* **324**: 1–8

Weidner N, Folkman J, Pozza F, Bevilacqua P, Allred EN, Moore DH, Meli S and Gasparini G (1992) Tumour angiogenesis: a new significant and independent prognostic indicator in early stage breast carcinoma. *J Natl Cancer Inst* **84**: 1875–1887

Weidner N, Carroll PR, Flax J, Blumenfeld W and Folkman J (1993) Tumour angiogenesis correlates with metastasis in invasive prostate carcinoma. *Am J Pathol* **143**: 401–409

Wiggins DL, Granai CO, Steinhoff MM and Calabresi P (1995) Tumour angiogenesis as a prognostic factor in cervical carcinoma. *Gynecol Oncol* **56**: 353–356

Wizigmann-Voos S, Breier G, Risau W and Plate KH (1995) Up-regulation of VEGF and its receptors in von Hippel-Lindau disease-associated and sporadic hemangioblastomas. *Cancer Res* **55**: 1358–1364

Woodman CBJ, Rollason T, Ellis J, Tierney R, Wilson S and Young L (1996) Human papilloma virus infection and risk of epithelial abnormalities of the cervix. *Br J Cancer* **73**: 553–556

© Cancer Research Campaign 1997